# Design, Microstructure and Mechanical Properties of Cast Medium Entropy Aluminium Alloys

**DOI:** 10.1038/s41598-019-43329-w

**Published:** 2019-05-01

**Authors:** Jon Mikel Sanchez, Iban Vicario, Joseba Albizuri, Teresa Guraya, Eva Maria Acuña

**Affiliations:** 1 Tecnalia Research & Innovation, Department of Foundry and Steel Making, Derio, 48160 Spain; 2 Faculty of Engineering of Bilbao (UPV/EHU), Department of Mechanical Engineering, Bilbao, 48013 Spain; 3 Faculty of Engineering of Bilbao (UPV/EHU), Department of Mining & Metallurgical Engineering and Materials Science, Bilbao, 48013 Spain; 4 Leartiker, Department of Mechanical Characterization, Markina-Xemein, 48270 Spain

**Keywords:** Materials chemistry, Mechanical engineering, Metals and alloys

## Abstract

In this work, the design, microstructures and mechanical properties of five novel non-equiatomic lightweight medium entropy alloys were studied. The manufactured alloys were based on the Al_65_Cu_5_Mg_5_Si_15_Zn_5_X_5_ and Al_70_Cu_5_Mg_5_Si_10_Zn_5_X_5_ systems. The formation and presence of phases and microstructures were studied by introducing Fe, Ni, Cr, Mn and Zr. The feasibility of CALPHAD method for the design of new alloys was studied, demonstrating to be a good approach in the design of medium entropy alloys, due to accurate prediction of the phases, which were validated via X-ray diffraction and scanning electron microscopy with energy dispersive spectroscopy. In addition, the alloys were manufactured using an industrial-scale die-casting process to make the alloys viable as engineering materials. In terms of mechanical properties, the alloys exhibited moderate plastic deformation and very high compressive strength up to 644 MPa. Finally, the reported microhardness value was in the range of 200 HV_0.1_ to 264 HV_0.1_, which was two to three times higher than those of commercial Al alloys.

## Introduction

Traditional alloys are based on a main element with additional elements alloyed to obtain the properties required for a specific industrial application. Therefore, a knowledge of alloys near the corners of a multicomponent phase diagram is well developed, with much less knowledge of alloys at the centre of the phase diagram^1^. The traditional alloy strategy has been very restrictive in exploring the full range of possible alloys, because there are many more compositions at the centre of a multicomponent phase diagram than at the corners.

To overcome the above concerns, High Entropy Alloys (HEAs) and equiatomic multicomponent alloys were proposed respectively by Yeh *et al*.^2^ and Cantor *et al*.^3^ in 2004. Unlike traditional alloys, HEAs or equiatomic multicomponent alloys were composed of five or more metallic elements in equimolar or near-equimolar ratios. The basic principle behind the new alloy strategy was to promote the formation of solid solution phases, avoiding the formation of brittle intermetallic compounds (ICs).

Yeh *et al*. proposed a classification of the alloys in function of the configurational entropy (ΔS_conf_). The alloys were classified as HEAs when their ΔS_conf_ at a random state was higher than 1.5 R (R being the gas constant), regardless of whether they are single-phase or multiphase at room temperature (RT). Alloys were classified as Medium Entropy Alloys (MEAs), when the values of their ΔS_conf_ were in the range from 1R to 1.5 R. Finally, some commercial alloys such as 7075 aluminium alloys or AZ91D were classified as low entropy alloys, its ΔS_conf_ is less than 1R^4^_._ Although, the initial publications focused on single-phase HEAs because of their excellent properties^5^, some multiphase and non-equiatomic HEAs also demonstrated to possess excellent mechanical and physical properties^6–8^. Thus, the term Complex Concentrated Alloys (CCAs) was introduced for multiphase HEAs. For the sake of simplicity during the present work, the term HEAs is used for single phase SS when ΔS_conf_ ≥ 1.5 R. The term CCAs is used for multiphase alloys when ΔS_conf_ ≥ 1.5 R. Finally, alloys are named MEAs when 1R ≤ ΔS_conf_ ≤ 1.5 R.

The most commonly used melting techniques to manufacture HEAs, MEAs and CCAs are vacuum arc melting and vacuum induction melting^9^. These techniques are basically based on melting in a protective atmosphere and casting in a water refrigerated copper mould. Multiple repetitive melting and solidification are often performed to guarantee the chemical homogeneity of the alloys. The principles of manufacturing HEAs were studied by Kumar *et al*.^10^ and Jablonski *et al*.^11^. Despite the expensive casting process, HEAs and most CCAs usually possess poor liquidity and castability, and considerable compositional inhomogeneity. The reason is that they contain multiple elements with high concentrations. In addition, the melting point of some elements (Cr, Fe, Ti…) can be higher than the boiling point of other alloying elements like Mg, Li or Zn, which can lead to evaporation losses and casting defects. Thus, the industrial scale manufacturing of HEAs, MEAs and CCAs has become a new challenge due to the high complexity of the process.

Inspired by HEAs, Raabe *et al*. developed novel steels based on high ΔS_conf_ for stabilizing a single-phase SS matrix^12^. The ΔS_conf_ of these alloys are between 1R and 1.5 R, which means that they can be classified as MEAs. They studied several alloys based on the Fe-Mn-Al-Si-C and Fe-Mn-Al-C systems with a Fe concentration between 41–70 at.%. The mechanical properties of the studied alloys were superior compared to those of many conventional austenitic steels. Subsequently, numerous works have been carried out based on MEAs with Fe as main element^13–18^. A similar design strategy was employed by Laws *et al*., creating a range of high entropy brasses and bronzes. The Cu-Mn-Ni ternary system with the addition of Al, Sn and Zn was explored. These alloys showed high compressive strength, ductility and hardness in comparison with standard commercial brasses^19^.

A great deal of research has been conducted on the development of MEAs with exceptional mechanical properties^20–28^, but few experiments have been carried out to discover novel lightweight MEAs (LWMEAs). Instead, excellent mechanical properties were often obtained on compression and hardness tests for studied LWMEAs. Mg_46_(MnAlZnCu)_54_ and Mg_50_(MnAlZnCu)_50_ alloys exhibited moderate hardness (178 HV-226 HV) and high compression strength (400 MPa–482 MPa) at RT, but they exhibited a brittle behaviour^29^. Equimolar MgMnAlZnCu alloy was also studied, which has similar mechanical properties but a higher density^30^. The Al-Li-Mg-Zn-Cu and Al-Li-Mg-Zn-Sn high and medium entropy systems were studied by Yang *et al*.^31^. All the alloys exhibited high strength, and the plastic strain of Al_80_Li_5_Mg_5_Zn_5_Sn_5_ and Al_80_Li_5_Mg_5_Zn_5_Cu_5_ MEAs reached up to 17 and 16% respectively. On the other hand, AlLiMgZnSn and AlLi_0.5_MgZn_0.5_Sn_0.2_ alloys exhibited very brittle behaviour, with plastic strain values below 1,2%. In another related study, Beak *et al*. reported the microstructure and compressive properties of Al_70_Mg_10_Si_10_Cu_5_Zn_5_ alloy at RT and 350 °C. An ultrasonic melt treatment was used to improve the ultimate compressive strength of as-cast alloy from 573 to 681 MPa^32^. The precipitation behaviour and the mechanical properties of Al-6Mg-9Si-10Cu-10Zn-3Ni (wt.%) alloy was investigated by Ahn *et al*.^33,34^. The Al-6Mg-9Si-10Cu-10Zn-3Ni alloy showed excellent mechanical properties when compared to some of the commercial Al alloys. Recently, a series of lightweight Al–Mg system entropic alloys containing Zn, Cu, and Si were studied by Shao *et al*.^35^. The fabricated alloys had high strength, with compressive strength exceeding 500 MPa at RT.

Recently, a new route to design non-equiatomic MEAs that contains one matrix element and several alloying elements was proposed. The range of matrix composition can be up to 66 at.%, 71 at.% and 73 at.% for quaternary, quinary and senary systems, respectively^36^. This simplified the complex design, manufacture and physical metallurgy of HEAs and most CCAs. In addition, MEAs also have the advantage of benefiting from the “four core effect” proposed for HEAs^37^. Despite the physical metallurgy and composition of MEAs is not as complex as HEAs or CCAs, the design of MEAs is still a challenge. To date, CALPHAD method has demonstrated to be the most reliable method for the study and the design of HEAs and CCAs^38–40^. However, further work is still needed on the design of MEAs to make them viable as engineering materials.

Motivated by the above concerns, and because of the great potential that LWMEAs offer for widespread applications, the design, microstructures and mechanical properties of five novel non-equiatomic MEAs based on the Al_65_Cu_5_Mg_5_Si_15_Zn_5_X_5_ and Al_70_Cu_5_Mg_5_Si_10_Zn_5_Y_5_ (X = Fe, Ni and Y = Cr, Mn, Zr, in at.%) systems were studied. The alloys were designed following the application-based redesign strategy^41^. They were designed in a framework of five elements and a variable high melting point transition metal was added to each alloy. The elements were selected on the basis that Al-Si alloys are the most common casting alloys in the automotive industry. The major alloying elements used in Al-Si alloys are Cu, Mg, Ni and Zn, as their addition improve the mechanical and physical properties of the alloys^42^. It should be noted that the use of expensive or scarce elements was avoided, and that they were fabricated by an industrial scale die casting process. They were designed by Thermo-Calc to achieve a ductile α-Al matrix reinforced with different ICs to overcome the weaknesses (the decrease of their mechanical properties at high temperatures, poor wear behaviour and low strength and hardness) of commercial Al alloys.

## Results

### Design of medium entropy alloys

Following the previously mentioned new route to design non-equiatomic MEAs, the range of matrix composition was defined between 65 at.% and 70 at.%. Thus, from above mentioned classification based on the ranges of ∆S_conf_, alloys can be divided in low, medium and high entropy alloys. According to Boltzmann’s hypothesis, for a n-element multicomponent alloy at a random state, ΔS_conf_ can be calculated by the following equation:

1 $${{\rm{\Delta }}S}_{{\rm{conf}}}=-\,{\rm{R}}{\sum }_{{\rm{i}}}^{{\rm{n}}}{{\rm{X}}}_{{\rm{i}}}{{\rm{lnX}}}_{{\rm{i}}}$$

where R is the constant of gases (8,314 J. (mol.K)^−1^), X_i_ is the fraction of atom of element “i” and “n” is the total number of elements. Based on Eq. (1), the values of ΔS_conf_ were 1,2 R J.mol/K for Al_65_Cu_5_Fe_5_Mg_5_Si_15_Zn_5_ and Al_65_Cu_5_Mg_5_Ni_5_Si_15_Zn_5_ and 1,1 R J.mol/K for Al_70_Cr_5_Cu_5_Mg_5_Si_10_Zn_5_, Al_70_Cu_5_Mg_5_Mn_5_Si_10_Zn_5_ and Al_70_Cu_5_Mg_5_Si_10_Zn_5_Zr_5_. Thus, all the studied alloys were classified as MEAs since their ΔS_conf_ values are between 1R and 1.5 R.

**Figure 1:**
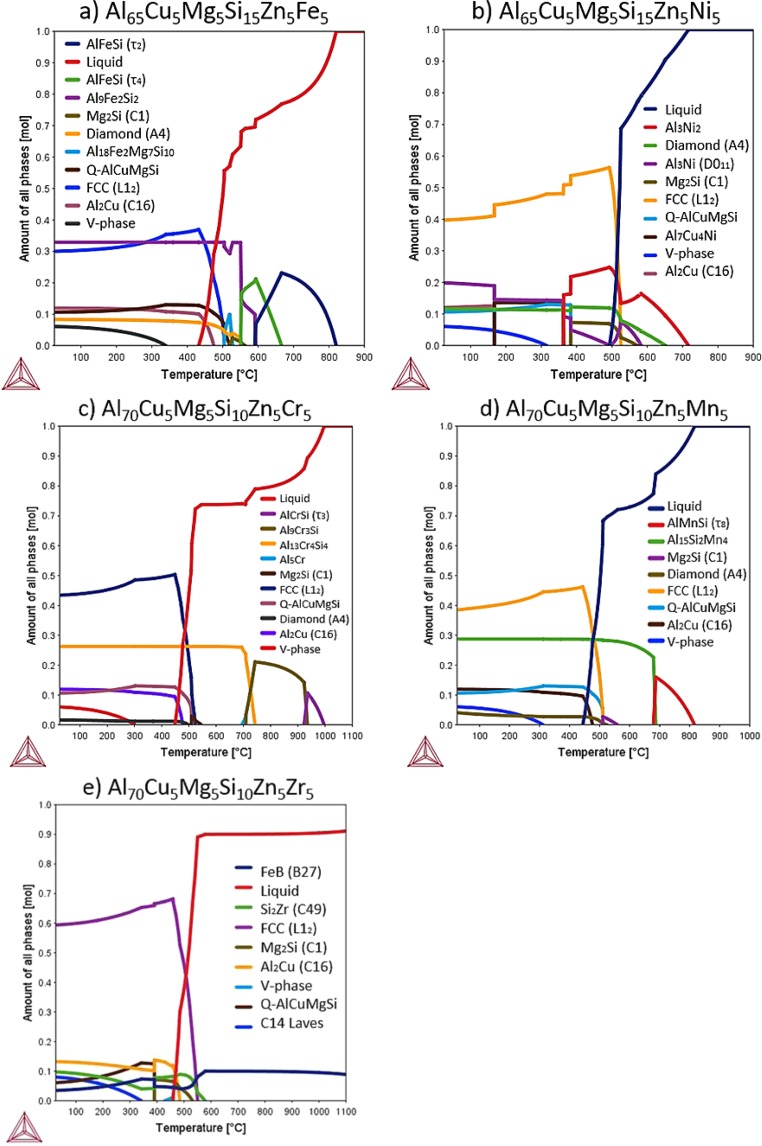
Amount of all phases VS temperature diagrams of designed alloys (**a**) Al_65_Cu_5_Fe_5_Mg_5_Si_15_Zn_5_, (**b**) Al_65_Cu_5_Mg_5_Ni_5_Si_15_Zn_5_, (**c**) Al_70_Cr_5_Cu_5_Mg_5_Si_10_Zn_5_, (**d**) Al_70_Cu_5_Mg_5_Mn_5_Si_10_Zn_5_ and (**e**) Al_70_Cu_5_Mg_5_Si_10_Zn_5_Zr_5_; using Thermo-Calc software with TCAL5 database.

In Fig. 1, the thermodynamic calculations of equilibrium phases as a function of temperature were calculated using Thermo-Calc software and TCAL5 database^43^. According to previous works^44–46^, the use of this database in Al-based HEAs and CCAs shows a good agreement with the experimental results. It should be noted that calculations were made for homogeneous alloys and not considering impurities, casting defects or oxides formed during the casting process. Moreover, it is well known that casting is not an equilibrium process. So, the studied alloys are not supposed to be in total thermodynamic equilibrium. The phase diagrams in Fig. 1 predicted at least the formation of major FCC (L1_2_), Al_2_Cu (C16), Diamond (A_4_), Q-AlCuMgSi and V-phase (Mg_2_Zn_11_) in all the alloys at RT, except in Al_70_Cu_5_Mg_5_Si_10_Zn_5_Zr_5_ alloy. FCC (L1_2_) refers to an ordered FCC phase closely related to the L1_2_ structures (Strukturbericht notation). The disordered structure of FCC (A1) solution transformed into ordered FCC (L1_2_), due to the complex composition of the alloys. The V-phase, which precipitated from the FCC solid solution, was the last precipitated phase in all the alloys, and the precipitation range was about 300 °C.

Figure 1(a) shows the equilibrium phase mole fraction of Al_65_Cu_5_Fe_5_Mg_5_Si_15_Zn_5_ alloy as a function of temperature. The phase diagram also predicted the formation of Al_9_Fe_2_Si_2_ (also known as β-Al_4.5_FeSi) at RT. The liquidus and solidus temperatures were 817 °C and 461 °C, respectively. Below solidus temperature, only V-phase was precipitated, at 329 °C. Thus, from CALPHAD calculations Al_65_Cu_5_Fe_5_Mg_5_Si_15_Zn_5_ alloy consisted of an ordered FCC solid-solution (30%) and five ICs (70%) at RT, with the main phase being Al_9_Fe_2_Si_2_ (33%). Figure 1(b) shows the phase diagram of Al_65_Cu_5_Mg_5_Ni_5_Si_15_Zn_5_ alloy as a function of temperature. The phase diagram predicted a similar phase equilibrium that is close to that obtained previously in Fig. 1(a) at RT. But with the difference of obtaining Al_3_Ni (D0_11_) instead of Al_9_Fe_2_Si_2_ phase. The liquidus and solidus temperatures were 716 °C and 488 °C, respectively. Some qualitative and quantitative phase transformations were calculated below solidus temperature. The Al_3_Ni_2_ phase, which was the major IC near liquidus temperature disappeared at 362 °C. The Q-phase was expected to precipitate at 382 °C, indicating that the Cu absorption of this phase led to the transformation of Al_3_Ni_2_ into an Al_3_Ni. The phase constitution of Al_3_Ni phase was (Al,Ni)_0.75_Ni_0.25_ and did not admit Cu. Instead, Al_3_Ni_2_ was calculated with a phase constitution of (Al, Si)_3_(Ni,Cu)_2_(Ni)_1_. So, the alloy consisted of a major ordered FCC solid-solution (40%) and five ICs (60%) at RT, with the main IC being Al_3_Ni (20%). Figure 1(c) shows the equilibrium phase mole fraction of Al_70_Cr_5_Cu_5_Mg_5_Si_10_Zn_5_ alloy as a function of temperature. The phase diagram also predicted the formation Al_13_Cr_4_Si_4_ at RT. The liquidus and solidus temperatures were 997 °C and 450 °C, respectively. The V-phase was stable at temperatures below 300 °C. From thermodynamic calculations in Fig. 1(c), it can be predicted that Al_70_Cr_5_Cu_5_Mg_5_Si_10_Zn_5_ alloy consist of an ordered FCC solid-solution (43%) and five ICs (57%) at RT, with the main IC being Al_13_Cr_4_Si_4_ (26%). The amount of this compound was stable below 692 °C. Figure 1(d) shows the equilibrium phase mole fraction of Al_70_Cu_5_Mg_5_Mn_5_Si_10_Zn_5_ alloy as a function of temperature. The diagram was closely related to the phase diagram in Fig. 1(c). The diagram also predicted the above-mentioned phases and the Al_15_Si_2_Mn_4_ compound at RT. The liquidus and solidus temperatures were 817 °C and 447 °C, respectively. From thermodynamic calculations in Fig. 1(d), it can be predicted that Al_70_Cu_5_Mg_5_Mn_5_Si_10_Zn_5_ alloy consisted of an ordered FCC solid-solution (39%) and five ICs (61%) at RT, with the main IC being Al_15_Si_2_Mn_4_ (29%). The amount of this compound was stable bellow 681 °C. Figure 1(e) shows the equilibrium phase mole fraction of Al_70_Cu_5_Mg_5_Si_10_Zn_5_Zr_5_ alloy as a function of temperature. The phase diagram is significantly more complex compared with previously calculated diagrams in Fig. 1(a–d). The phase diagram predicted the formation of major FCC (L1_2_), Al_2_Cu (C16), Q-AlCuMgSi, Si_2_Zr (C49), C14 Laves (Zn_2_Mg) phase and FeB (B27) at RT. The phase composition of FeB phase was (Zr)(Si, Zn). The liquidus temperature was over 1100 °C and solidus temperature was 454 °C. Many phase transformations were calculated below solidus temperature, resulting in a microstructure of an ordered FCC solid-solution (60%) and five ICs (40%) at RT, with the main IC being Al_2_Cu (13%).

### Microstructure characterization

The overall bulk composition of the alloys was estimated using scanning electron microscopy (SEM) equipped with an energy dispersive X-ray spectrometry (EDS) on large areas. At least three random measurements were made, and the overall values are presented in Table 1. The obtained results showed good approximations to target compositions of the alloys, which demonstrated that the manufacturing process was successfully performed, although oxidation was detected by SEM-EDS.

**Table 1:** Chemical composition in at.% of the manufactured alloys measured by SEM-EDS.

Nominal Composition	Al	Mg	Si	Zn	Cu	Fe	Ni	Cr	Mn	Zr	O
Al_65_Cu_5_Fe_5_Mg_5_Si_15_Zn_5_	64	6	13	6	5	4					2
Al_65_Cu_5_Mg_5_Ni_5_Si_15_Zn_5_	64	7	12	5	4		5				3
Al_70_Cr_5_Cu_5_Mg_5_Si_10_Zn_5_	73	5	7	6	3			4			2
Al_70_Cu_5_Mg_5_Mn_5_Si_10_Zn_5_	66	6	11	6	4				4		3
Al_70_Cu_5_Mg_5_Si_10_Zn_5_Zr_5_	66	7	9	6	7					3	2

**Figure 2:**
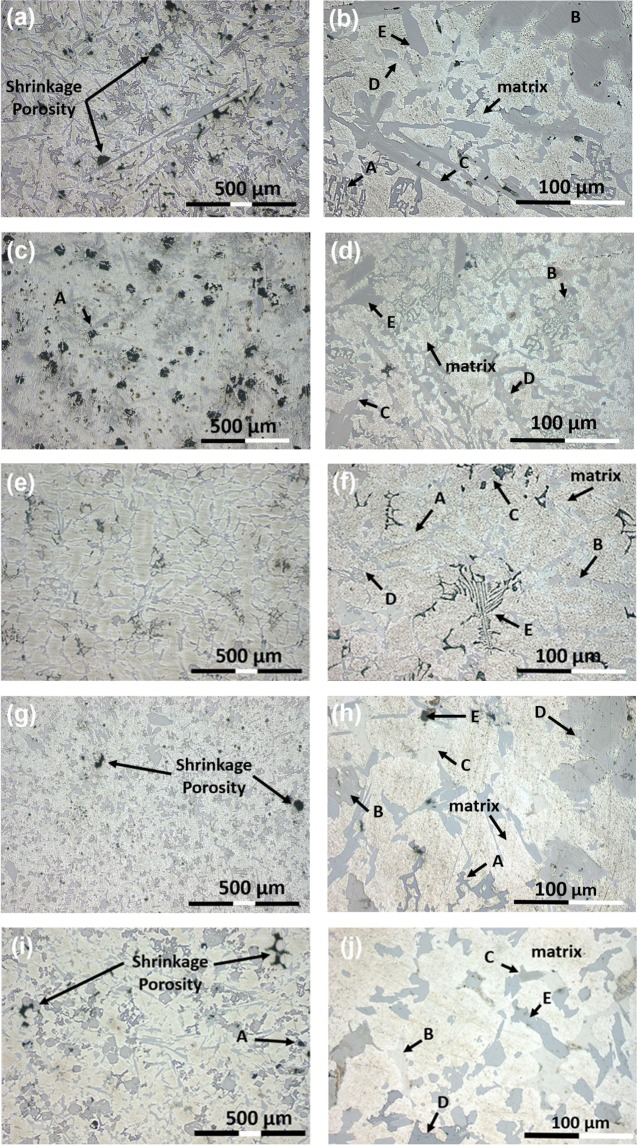
OM images of (**a**,**b**) Al_65_Cu_5_Fe_5_Mg_5_Si_15_Zn_5_, (**c**,**d**) Al_65_Cu_5_Mg_5_Ni_5_Si_15_Zn_5_, (**e**,**f**) Al_70_Cr_5_Cu_5_Mg_5_Si_10_Zn_5_, (**g**,**h**) Al_70_Cu_5_Mg_5_Mn_5_Si_10_Zn_5_ and (**i**,**j**) Al_70_Cu_5_Mg_5_Si_10_Zn_5_Zr_5_ manufactured alloys.

The optical microscopy (OM) image of Al_65_Cu_5_Fe_5_Mg_5_Si_15_Zn_5_ alloy is shown in Fig. 2(a). The microstructure of the alloy revealed that shrinkage porosity was distributed near the plate-like phase. This defect was caused by metal reducing its volume during solidification, and its inability to feed shrinkage around complex morphology of the phase. A magnified OM image is shown in Fig. 2(b), where a mixture of different phases and brightest matrix phase were observed. At least five phases (A, B, C, D and E) with different contrasts were observed. In Fig. 2(c), dark irregular blocky-shape phase (A) were observed in the microstructure of Al_65_Cu_5_Mg_5_Ni_5_Si_15_Zn_5_ alloy. The magnified OM image of the alloy is shown in Fig. 2(d), a mixture of different phases (B, C, D and E) and a brightest matrix phase were observed. In Fig. 2(e), the complex dendritic structure of Al_70_Cr_5_Cu_5_Mg_5_Si_10_Zn_5_ alloy is shown, which was divided by net-like interdendritic structure and different phases. The magnified OM image is shown in Fig. 2(f), the microstructure also showed a mixture of different phases (A, B, C, D and E) and brightest matrix phase. In Fig. 2(g), shrinkage porosity was observed in the microstructure of Al_70_Cu_5_Mg_5_Mn_5_Si_10_Zn_5_ alloy. The magnified OM image of Al_70_Cu_5_Mg_5_Mn_5_Si_10_Zn_5_ is shown in Fig. 2(h), the image revealed that the microstructure was composed of at least five phases (A, B, C, D and E) and brightest matrix phase. In Fig. 2(i), shrinkage porosity was observed in the microstructure of Al_70_Cu_5_Mg_5_Si_10_Zn_5_Zr_5_ alloy. The OM image also showed the formation of dark irregular blocky-shape phase (A), but these were much smaller in size than previously observed phase in Fig. 2(c). The magnified OM image is shown in Fig. 2(j), a mixture of randomly distributed phases (B, C, D and E) were observed in the matrix.

According to OM images in Fig. 2, a multiphase character can be expected for the manufactured alloys. The different images confirmed a heterogeneous microstructure composed of at least five phases and major FCC matrix. So, CALPHAD thermodynamic modelling successfully predicted the number of constituent phases at RT.

**Figure 3:**
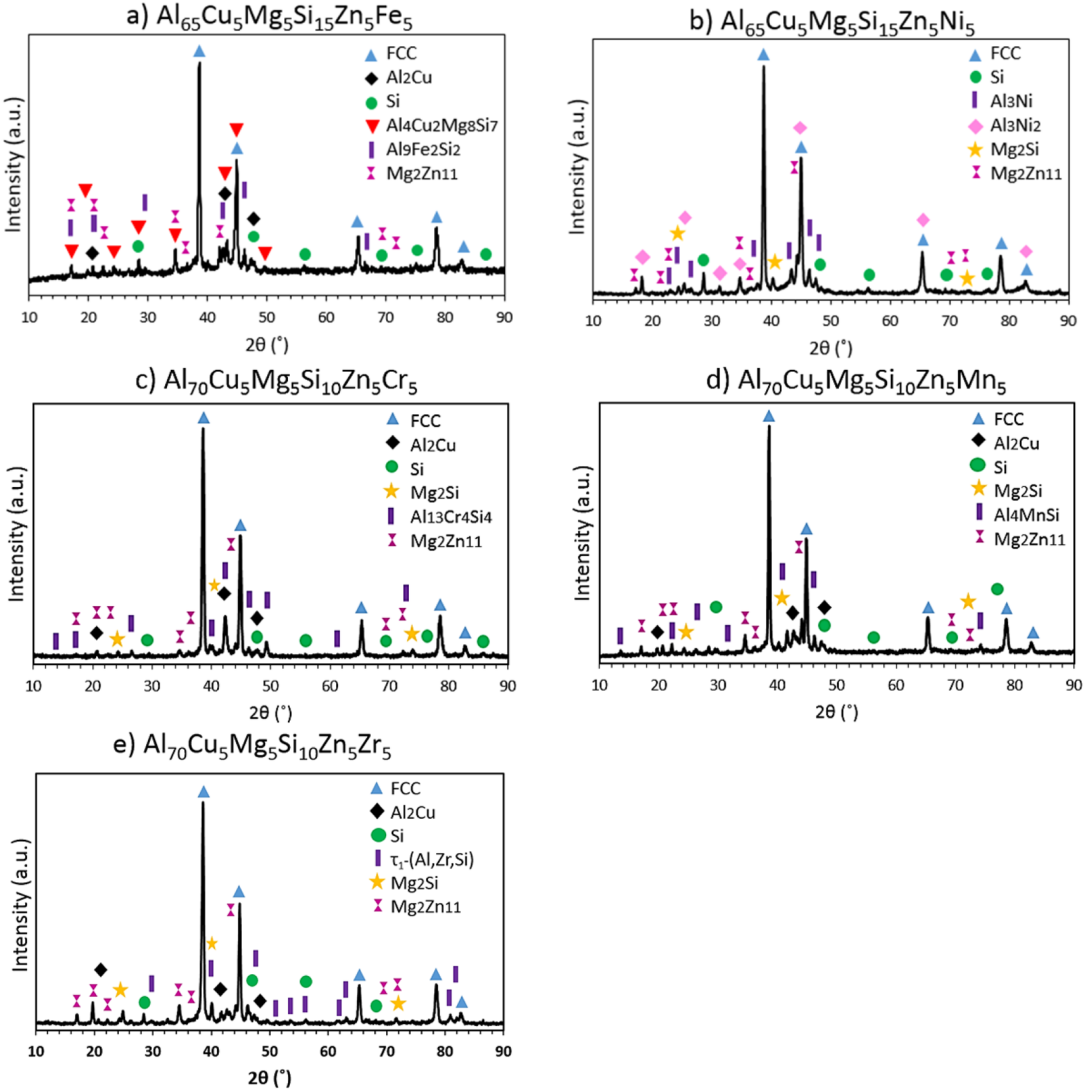
XRD diffraction patterns of the (**a**) Al_65_Cu_5_Fe_5_Mg_5_Si_15_Zn_5_, (**b**) Al_65_Cu_5_Mg_5_Ni_5_Si_15_Zn_5_, (**c**) Al_70_Cr_5_Cu_5_Mg_5_Si_10_Zn_5_, (**d**) Al_70_Cu_5_Mg_5_Mn_5_Si_10_Zn_5_ and (**e**) Al_70_Cu_5_Mg_5_Si_10_Zn_5_Zr_5_ as-cast alloys.

The XRD patterns in Fig. 3 showed at least the formation of FCC solid solution (Space Group = 225), Si (S.G. = 227) and V-Mg_2_Zn_11_ (S.G. = 218) in all the alloys. The XRD pattern in Fig. 3(a) also showed the formation of Al_2_Cu (S.G. = 140), Al_4_Cu_2_Mg_8_Si_7_ (S.G. = 174) and Al_9_Fe_2_Si_2_ phase (S.G. = 14) in Al_65_Cu_5_Fe_5_Mg_5_Si_15_Zn_5_ alloy. The experimental results obtained by XRD technique and CALPHAD simulation in Fig. 2(a) showed a good agreement. Figure 3(b) detailed the XRD pattern of Al_65_Cu_5_Mg_5_Ni_5_Si_15_Zn_5_ alloy. The pattern also showed the formation of Al_3_Ni (S.G. = 62), Al_3_Ni_2_ (S.G. = 164) and Mg_2_Si (S.G. = 225). Thus, FCC, Si, V-Mg_2_Zn_11_ and Al_3_Ni predicted phases were observed, but Al_2_Cu and Al_4_Cu_2_Mg_8_Si_7_ phases were not indexed. The XRD pattern showed the formation of Al_3_Ni_2_ and Mg_2_Si compounds at RT, but these phases were predicted at temperatures above 358 °C and 382 °C, respectively. Figure 3(c) detailed the XRD pattern of Al_65_Cu_5_Mg_5_Ni_5_Si_15_Zn_5_ alloy. The XRD pattern also showed the formation of Mg_2_Si and Al_13_Cr_4_Si_4_ (S.G. = 216) phases. The other indexed phases showed good agreement with CALPHAD calculations in Fig. 2(c).

Figure 3(d) detailed the XRD pattern of Al_70_Cu_5_Mg_5_Mn_5_Si_10_Zn_5_ alloy. The diagram is very similar to the diagram represented in Fig. 3(c), but Al_4_MnSi (S.G. = 194) phase was observed instead of Al_13_Cr_4_Si_4_ indexed in Fig. 3(c). In this case, the formation of the Mg_2_Si phase mentioned above was also observed. Figure 3(e) detailed the XRD pattern of Al_70_Cu_5_Mg_5_Si_10_Zn_5_Zr_5_ alloy. The diagram showed similar diffraction peaks to those observed in Fig. 3(c,d). But, τ_1_-(Al,Zr,Si) (S.G. = 194) phase was indexed instead of Al_13_ Cr_4_ Si_4_ and Al_4_MnSi phases. The experimental result showed some discrepancies with thermodynamics results in Fig. 2(e). Phase diagram predicted the formation of FCC, Al_2_Cu, Q-AlCuMgSi, Si_2_Zr, C14 Laves (Zn_2_Mg) and FeB phases at RT. Thus, Si_2_Zr, C14 Laves (Zn_2_Mg) and FeB phases were not observed by XRD.

**Figure 4:**
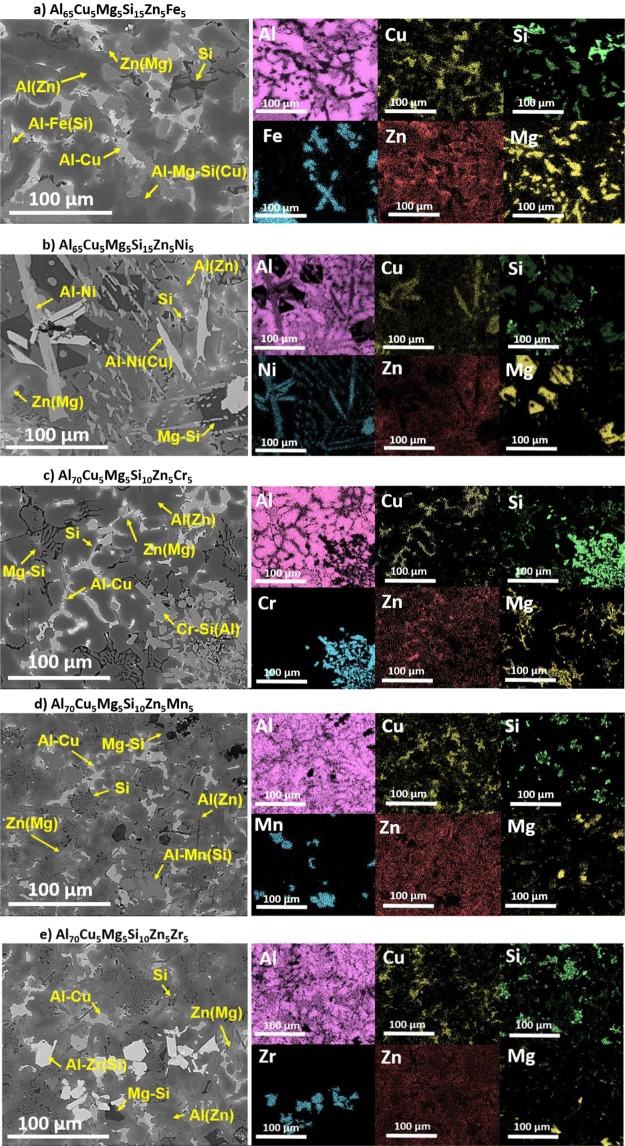
SEM image and EDS elemental mapping of (**a**) Al_65_Cu_5_Fe_5_Mg_5_Si_15_Zn_5_, (**b**) Al_65_Cu_5_Mg_5_Ni_5_Si_15_Zn_5_, (**c**) Al_70_Cr_5_Cu_5_Mg_5_Si_10_Zn_5_, (**d**) Al_70_Cu_5_Mg_5_Mn_5_Si_10_Zn_5_ and (**e**) Al_70_Cu_5_Mg_5_Si_10_Zn_5_Zr_5_ as-cast alloys.

To reveal the qualitative chemical compositions of the regions, EDS mappings were obtained in Fig. 4. For a better understanding of the maps, oxygen was neglected due to the insignificant amount that it represented in the composition of the alloys. Figure 4(a) showed the complex microstructure of the Al_65_Cu_5_Fe_5_Mg_5_Si_15_Zn_5_ alloy. The matrix is rich in Al with a small amount of Zn. This region corresponds to FCC phase detected by XRD. In the remaining space, Al(Cu)-rich region was distinguished, which agrees well with Al_2_Cu compound obtained by XRD measurements and thermodynamic calculations. Si was segregated in two different regions. The first one, it was mainly composed of Si and it was surrounded by the matrix. The second region, although rich in Si, was also composed of Al and Fe. It presented a plate-like morphology and it was related to Al_9_Fe_2_Si phase, this phase was not distributed homogeneously in Fig. 4(a). The brightest region was mainly composed of Zn and a small amount of Mg. This region was related to V-Mg_2_Zn_11_ phase. Finally, Al-Mg-Si(Cu)-rich region was distinguished, and this region corresponded to the above defined Q-phase.

In Fig. 4(b), two types of needle-like regions and dark blocky-shape precipitates are distributed in the matrix of Al_65_Cu_5_Mg_5_Ni_5_Si_15_Zn_5_ as-cast alloy. The matrix was rich in Al and Zn, with a small amount of Cu and Si. From EDS mapping, the longest needle-like region was composed of Al and Ni. This was consistent with the phase composition of Al_3_Ni compound predicted by Thermo-Calc. The second needle-like region presented similar morphology, but it was found that quite a few concentrations of Cu were dissolved in the region. This Cu absorption leaded to the formation of Al_3_Ni_2_ phase, which was not supposed to be in thermodynamic equilibrium at RT. The phase diagram in Fig. 1(b) shifted the formation of Al_3_Ni phase at temperatures below 380 °C. At this temperature, Al_3_Ni and Q-AlCuMgSi phases precipitated from Al_3_Ni_2_. In the XRD pattern of Fig. 3(b), Al_2_Cu and Q-AlCuMgSi phases were not observed. Therefore, according to the observations, Cu got trapped in Al_3_Ni_2_ phase, avoiding the formation of Al_2_Cu and Q-AlCuMgSi phases. In Al_65_Cu_5_Mg_5_Ni_5_Si_15_Zn_5_ alloy, Si was also segregated in two different regions, forming Si-rich phase and Mg-Si-rich blocky-morphology region. This blocky region corresponded to previously defined Mg_2_Si phase. The brightest region was mainly composed of Zn and a small amount of Mg. This region was related to V-Mg_2_Zn_11_ phase defined by XRD.

Figure 4(c) shows Al-rich dendritic region that was enriched with Zn. The Fig. 4(c) also shows that Al_2_Cu compound was precipitated in the interdendritic space. There were many non-uniform particles dispersed in the microstructure. These particles presented irregular blocky-form and were composed of Al, Cr and Si. These particles were correlated to Al_13_Cr_4_Si_4_ phase. The brightest region in the interdendritic space, it was mainly composed of Zn and a small amount of Mg. This region was corresponded to Mg_2_Zn_11_ phase. Finally, the Chinese script region was composed of the matrix and Mg-Si-rich skeleton-like precipitates. This region presented different morphology than the one shown in Fig. 4(a), but quite similar qualitative elemental composition.

In Fig. 4(d), the as-cast microstructure consists of a dominant set of coarse Al(Zn) dendrites with a minor set of bright precipitates randomly distributed in the dark background. The dendritic structure was rich in Al and is also composed of small amounts of Cu and Zn. The interdendritic space was rich in Al and Cu, corresponding to Al_2_Cu phase. The eutectic region was composed of Si-rich particles precipitated in Al-rich dendrites. The Zn(Mg)-rich region was correlated to V-Mg_2_Zn_11_ phase. A region composed of Al, Mn and Si with blocky-morphology was distinguished, and this region was correlated to Al_4_MnSi phase defined by XRD in Fig. 3(d). Finally, an irregular dark blocky-shape Mg-Si-rich region was distinguished. This region corresponded to Mg_2_Si phase defined by XRD.

In Fig. 4(e) Al(Zn)-rich dendrites and Al-Cu-rich interdendritic regions are shown. These regions corresponded to FCC and Al_2_Cu phases respectively. The eutectic region was composed of Si-rich particles precipitated in Al(Zn) dendrites. This eutectic region was also observed in Al_70_Cu_5_Mg_5_Mn_5_Si_10_Zn_5_ alloy. The darkest region in SEM image corresponded to Mg-Si-rich region, correlated to Mg_2_Si phase. The brightest region was composed of Al, Si and Zr, and it presented blocky-morphology, and was not homogenously distributed in the microstructure of the alloy. So, the formation of τ_1_-(Al,Zr,Si) phase was confirmed in Fig. 4(a). The chemical composition of Al-Zr(Si)-rich phases obtained in the present study was far from the stoichiometric composition of the Si_2_Zr and FeB ((Zr)(Si,Zn)) phases predicted by Thermo-Calc. The reason for the discrepancies between CALPHAD and experimental results was that although TCAL5 database contains assessments of 87 ternaries^43^, the ternary Al-Si-Zr system is not assessed. Therefore, the predictions of binary compounds of Si-Zr in Fig. 1(d) may not have been entirely correct. Finally, the Zn(Mg)-rich region was correlated to V- Mg_2_Zn_11_ phase. The phase diagram in Fig. 2(e) shows that the formation sequence of the phases was FeB, Si_2_Zr, FCC, Mg_2_Si, Al_2_Cu, V-phase, Q-phase and finally, C14 Laves phase. Thus, the observation of Mg_2_Si phase in the diffraction diagram meant that Q-phase could not be formed from Mg_2_Si. Subsequently, C14 Laves phase was not precipitated from Q-phase. Thus, the experimental observations of the Al_70_Cu_5_Mg_5_Si_10_Zn_5_Zr_5_ as-cast alloy corresponded only partially to the predictions in Fig. 2(e).

The Mg_2_Si phase was not expected to be in thermodynamic equilibrium at RT. But XRD and SEM-EDS results clearly showed that Mg_2_Si phase was formed in Al_65_Cu_5_Mg_5_Ni_5_Si_15_Zn_5_, Al_70_Cr_5_Cu_5_Mg_5_Si_10_Zn_5_, Al_70_Cu_5_Mg_5_Mn_5_Si_10_Zn_5_ and Al_70_Cu_5_Mg_5_Si_10_Zn_5_Zr_5_ alloys. So, the quantitatively chemical compositions of Mg-Si-rich regions in Fig. 2(b–e) were analysed using SEM-EDS in Table 2.

**Table 2:** Chemical compositions of the Mg-Si-rich regions in the as-cast alloys measured by SEM-EDS (at.%).

Alloy	Mg	Si	O	Al
Al_65_Cu_5_Mg_5_Ni_5_Si_15_Zn_5_	54	28	18	—
Al_70_Cr_5_Cu_5_Mg_5_Si_10_Zn_5_	41	23	24	12
Al_70_Cu_5_Mg_5_Mn_5_Si_10_Zn_5_	55	29	16	—
Al_70_Cu_5_Mg_5_Si_10_Zn_5_Zr_5_	55	30	15	

The compositions in Table 2 revealed that Mg_2_Si phase was oxidized, due to the high reactivity of Mg at the temperatures reached up during the melting. The chemical composition of Mg-Si-rich regions in Al_65_Cu_5_Mg_5_Ni_5_Si_15_Zn_5_, Al_70_Cu_5_Mg_5_Mn_5_Si_10_Zn_5_ and Al_70_Cu_5_Mg_5_Si_10_Zn_5_Zr_5_ phases was very similar. The phases presented typical blocky morphology of Mg_2_Si compound, as can be observed in Fig. 4(b,d,e). On the other hand, in Al_70_Cr_5_Cu_5_Mg_5_Si_10_Zn_5_ alloy, the absorption of a small amount (12 at.%) of Al modified the blocky morphology into a Chinese script morphology. But this was only confirmed in Al_70_Cr_5_Cu_5_Mg_5_Si_10_Zn_5_ alloy, and the reasons for this have not been determined. The Chinese script phase is preferred because it is less detrimental to mechanical properties.

### Mechanical properties

**Figure 5:**
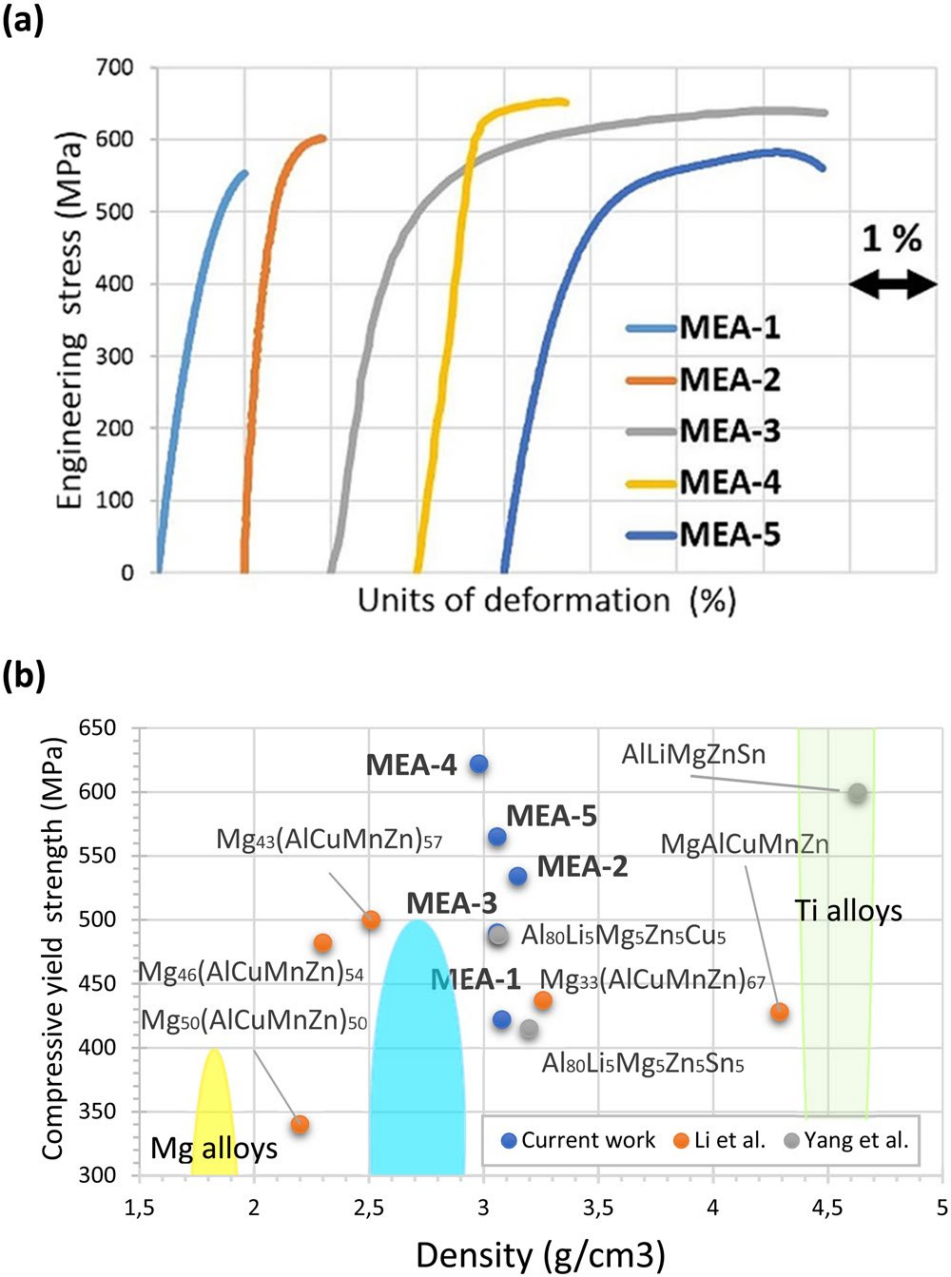
(**a**) Compressive engineering stress–strain curves of manufactured Al_65_Cu_5_Fe_5_Mg_5_Si_15_Zn_5_ (MEA-1), Al_65_Cu_5_Mg_5_Ni_5_Si_15_Zn_5_ (MEA-2), Al_70_Cr_5_Cu_5_Mg_5_Si_10_Zn_5_ (MEA-3), Al_70_Cu_5_Mg_5_Mn_5_Si_10_Zn_5_ (MEA-4) and Al_70_Cu_5_Mg_5_Si_10_Zn_5_Zr_5_ (MEA-5) MEAs at RT. (**b**) Materials property space for compressive yield strength vs density at RT of manufactured alloys, related works and conventional lightweight alloys.

Up to the present, compressive properties and Vickers’s microhardness are the most reported properties studied for MEAs, HEAs and CCAs. In Table 3, the density and compressive mechanical properties with the standard deviations of the manufactured MEAs were summarized. Figure 5(a) shows the engineering compressive stress-strain curves of the manufactured MEAs. In this study, the fracture strength and the plastic strain were defined as the maximum stress and the maximum deformation in the stress-strain curve under each testing condition. The experimental scatter was 2–3% for the maximum compressive deformation. The Al_65_Cu_5_Fe_5_Mg_5_Si_15_Zn_5_ and Al_65_Cu_5_Mg_5_Ni_5_Si_15_Zn_5_ alloys had high RT strength (σ_max_ = 482 MPa and σ_max_ = 594 MPa) but showed a true compressive fracture strain of only 1%. In the case of Al_65_Cu_5_Fe_5_Mg_5_Si_15_Zn_5_, the brittle behaviour is due to Al_9_Fe_2_Si_2_ phase, which is a well-known phase that decreases the ductility of the Al alloys. The entropy of the system (1,2 R) was not enough to avoid the formation of this undesirable phase. In Al_65_Cu_5_Mg_5_Ni_5_Si_15_Zn_5_ the brittle behaviour was attributed to the large size of the observed oxides in Fig. 2(c). The Al_70_Cr_5_Cu_5_Mg_5_Si_10_Zn_5_ alloy combines the best results in terms of ductility-strength. It was reported a σ_y_ of 490 MPa with a maximum nominal plastic strain of 6%. The microstructure of Al_70_Cr_5_Cu_5_Mg_5_Si_10_Zn_5_ alloy in Fig. 2(f) showed the formation of beneficial Chinese script phase instead of the brittle blocky Mg_2_Si phase. Moreover, the size and distribution of the main IC (Al_13_Cr_4_Si_4_) was less detrimental to the ductility, and porosity was not detected in Fig. 2(e). The Al_70_Cu_5_Mg_5_Mn_5_Si_10_Zn_5_ alloy exhibited very high compressive strength, but the deformation values dropped below 2%. The Al_70_Cu_5_Mg_5_Si_10_Zn_5_Zr_5_ alloy, which was designed to have the largest volume of ductile α-Al matrix, also exhibited plastic deformability and a very high compressive σ_y_ of 565 MPa. The maximum deformation reached up to 4%. Both alloys presented some shrinkage porosity.

**Table 3:** Density (ρ) and compressive mechanical properties of the manufactured alloys at RT.

Alloy	ρ (g/cm^3^)	σ_y_ (MPa)	σ_max_ (MPa)	ε_max_ (%)	E (GPa)	σ_max_/ρ
Al_65_Cu_5_Fe_5_Mg_5_Si_15_Zn_5_	3.08	422 ± 75	482 ± 98	1	88.7 ± 04	156
Al_65_Cu_5_Mg_5_Ni_5_Si_15_Zn_5_	3.15	534 ± 04	574 ± 32	1	107.8 ± 17	182
Al_70_Cr_5_Cu_5_Mg_5_Si_10_Zn_5_	3.06	490 ± 18	608 ± 30	6	78.4 ± 03	199
Al_70_Cu_5_Mg_5_Mn_5_Si_10_Zn_5_	2.98	622 ± 15	644 ± 13	2	114.1 ± 02	216
Al_70_Cu_5_Mg_5_Si_10_Zn_5_Zr_5_	3.06	565 ± 79	633 ± 42	4	105.1 ± 27	207

In terms of microhardness testing, the manufactured alloys also showed good performance. The average Vickers microhardness values with standard deviations are 235 ± 85 HV_0.1_, 260 ± 32 HV_0.1_, 200 ± 18 HV_0.1_, 264 ± 57 HV_0.1_ and 220 ± 37 HV_0.1_, respectively. No cracks were observed during indentation test, which means that all the alloys still possess the potential plastic deformability. Although some alloys presented poor ductility. Microstructure of Al_70_Cu_5_Mg_5_Mn_5_Si_10_Zn_5_ alloy, which showed the highest hardness value, exhibited the same influence on hardness as for strength. The second-phase strengthening mechanism of this alloy caused by the Al_4_MnSi phase was more pronounced, decreasing the ductility, but increasing the hardness and strength. The standard deviation of Al_65_Cu_5_Fe_5_Mg_5_Si_15_Zn_5_ and Al_70_Cu_5_Mg_5_Si_10_Zn_5_Zr_5_ alloys was significantly higher in terms of strength (Table 3) and hardness values. This is due to the large size and the not uniformly distributed regions in Fig. 4(a,e), Al-Fe(Si) and Al-Zr(Si), respectively.

A comparison of the strength and density of these MEAs with related works in the field of LWMEAs and commercial lightweight materials are given in Fig. 5(b). The materials in the present work are very well situated in the strength/density diagrams. The manufactured MEAs are superior in terms of strength/density to previously studied MEAs and most of conventional alloys. Furthermore, MEAs fill the gap between Al alloys, Mg alloys and Ti alloys.

## Discussion

In this work, five new lightweight non-equiatomic MEAs were studied, namely Al_65_Cu_5_Fe_5_Mg_5_Si_15_Zn_5_, Al_65_Cu_5_Mg_5_Ni_5_Si_15_Zn_5_, Al_70_Cr_5_Cu_5_Mg_5_Si_10_Zn_5_, Al_70_Cu_5_Mg_5_Mn_5_Si_10_Zn_5_ and Al_70_Cu_5_Mg_5_Si_10_Zn_5_Zr_5_. The experimental techniques revealed that all studied MEAs were a mixture of α-Al matrix and five intermetallic phases. The major intermetallic phase in all the alloys was mainly composed of Al-Fe, Al-Ni, Al-Cr, Al-Mn or Al-Zr. The present results show that it was difficult to form solid solutions phases in Al based Medium Entropy Alloys. The competition between enthalpy and entropy promoted the formation of ICs, due to the high negative mixing enthalpy of Al with transition metals. The role of each transition metal added to both systems was to promote the formation of the main IC, without affecting the formation of the remaining phases.

Some discrepancies between CALPHAD methodology and experimental values are clearly visible. The Q-AlCuMgSi phase only was experimentally observed in Al_65_Cu_5_Fe_5_Mg_5_Si_15_Zn_5_ alloy. The Mg_2_Si phase was not supposed to be in thermodynamic equilibrium at RT, but the peaks at 24°, 41° and 73° in the XRD patterns indicated the formation of this phase, instead of the predicted Q-AlCuMgSi. The phase diagrams in Fig. 2 showed that Q-AlCuMgSi precipitated from Mg_2_Si at 382 °C. Therefore, the experimental observation of Mg_2_Si instead of Q-AlCuMgSi phase at RT, suggested that Mg and Si were trapped in Mg_2_Si phase due to the oxidation of Mg, avoiding the precipitation of Q-AlCuMgSi. This observation did not contribute to the formation of V-Mg_2_Zn_11_ phase (which is composed of a small amount of Mg) since Mg precipitated from the FCC solid solution phase at temperatures below 300 °C. The oxidation of Mg was due the long-time of exposure of the molten alloy to high temperatures and the impurities in the alloying tablets. All the alloys reached to similar maximum temperatures during melting and were poured in the die in the same temperature range. But all the alloys exhibited oxidized Mg_2_Si phase, except Al_65_Cu_5_Fe_5_Mg_5_Si_15_Zn_5_ alloy, that was the only alloy in which only pure elements were used.

The Al_70_Cu_5_Mg_5_Si_10_Zn_5_X_5_ system exhibited the best mechanical properties. Specifically, Al_70_Cr_5_Cu_5_Mg_5_Si_10_Zn_5_ alloy showed the best mechanical properties in terms of strength-ductility. The main reason for that, was the transformation of the blocky morphology into a more desirable Chinese script morphology of Mg_2_Si phase, and the size and distribution of the Al_13_Cr_4_Si_4_ compound. In contrast to the rest of the alloys, with large ICs not uniformly distributed in their microstructure.

The casting process has shown that lightweight MEAs with high strength and high hardness can be adapted to large-scale industrial production. However, the melting process should be slightly adjusted to avoid shrinkage porosity and oxidization of Mg_2_Si phase. Oxidation of the Mg_2_Si phase can be avoided by using pure elements or mother alloys, but the use of briquettes should be avoided. Although the microstructure of the alloys presents some defects such as shrinkage porosity and oxides formation, these alloys have proven to have numerous possible applications, due to the viability of the industrial scale manufacturing and the obtained results in terms of mechanical properties. But the manufacturing method should be optimized to improve the ductility. Furthermore, the alloys were studied in the as-cast state, so the standard heat treatment for Al alloys could improve the moderate ductility of the alloys. As a further work, an exhaustive study of the mechanical properties, and especially the tribological properties of the as-cast and heat-treated alloys should be studied.

## Methods

### Design of the alloys

The software Thermo-Calc (v. 2017b, Thermo-Calc Software AB, Stockholm, Sweden)^47^ in conjunction with the TCAL5 thermodynamic database was used for calculations of the equilibrium phases as a function of temperature.

### Materials preparation

Experimental alloys were prepared in an induction furnace VIP-I (Inductotherm Corp., Rancocas, USA) in an alumina crucible using 99.95% pure Al, Cu, Fe, Mg, Si and Zn. Tablets of Al-Cr, Al-Mn, Al-Ni and Al-Zr containing 75 wt.% of Cr, 80 wt., 80 wt.% of Mn, 80 wt.% of Ni and 75 wt.% of Zr respectively were used. Approximately 4.5 kg were obtained for each alloy. The melting process can be divided into three stages. Firstly, Al and Si were placed at the bottom of the crucible to guarantee a bath base where the other elements were dissolved from highest to lowest melting point. In the second stage, the variable element of each alloy (Fe, Ni, Cr, Mn or Zr) was added to the molten alloy. The maximum temperature was reached at this stage. Finally, Cu, Zn and Mg were added respectively and held around 750 **°**C, at least 10 minutes to reach complete dissolution. Then, the melt was poured manually into a steel mould. The nominal liquidus temperatures obtained by Thermo-Calc, maximum temperatures reached up during the melting and casting temperatures are represented in Table 4.

**Table 4:** Nominal compositions (at.%), nominal liquidus temperatures (°C) obtained by Thermo-Calc, experimental maximum temperatures (°C) of the process and experimental casting temperatures (°C) of the manufactured alloys.

Nominal comp.	Liquidus	Maximum temp.	Casting temp.
Al_65_Cu_5_Fe_5_Mg_5_Si_15_Zn_5_	817	790	760
Al_65_Cu_5_Mg_5_Ni_5_Si_15_Zn_5_	716	785	759
Al_70_Cr_5_Cu_5_Mg_5_Si_10_Zn_5_	997	780	744
Al_70_Cu_5_Mg_5_Mn_5_Si_10_Zn_5_	817	830	750
Al_70_Cu_5_Mg_5_Si_10_Zn_5_Zr_5_	>1100	850	742

### Microstructural and elemental characterization

Several ingots of approximately 200 mm (length) × 80 mm (width) × 40 mm (thickness) were obtained for each alloy. The samples for optical microscopy (OM) and microhardness test were cut from the ingots and prepared according to standard metallographic procedures, by hot mounting in conductive resin, grinding, and polishing. The X-ray diffraction (XRD) equipment used to characterize the crystal structures of the alloys was a model D8 ADVANCE (BRUKER, Karlsruhe, Germany), with Cu Kα radiation, operated at 40 kV and 30 mA. The diffraction diagrams were measured at the diffraction angle 2θ, range from 10° to 90° with a step size of 0.01°, and 1.8 s/step. The powder diffraction file (PDF) database 2008 was applied for phase identification. The microstructure, the different regions and the averaged overall chemical composition of each sample were investigated by an optic microscope model DMI5000 M (LEICA Microsystems, Wetzlar, Germany) and a scanning electron microscope (SEM), equipped with an energy dispersive X-ray spectrometry (EDS) model JSM-5910LV (JEOL, Croissy-sur-Seine, France).

### Mechanical characterization

Cylindrical specimens for compression testing were machined from the ingots, with a diameter of 13 mm and a height of 26 mm, giving an aspect ratio of 1:2. Compression testing was performed using an MTS Insight 100 kN Extended Length (MTS Systems Corporation, Eden Prairie, USA) at RT with a strain rate of 0.001 s^−1^. For the accurate measurement of Young’s modulus, clip-on extensometer mounted on the specimens were used. At least five specimens were performed to ensure the repeatability. Vickers microhardness FM-700 model (FUTURE-TECH, Kawasaki, Japan) was employed on the polished sample surface using a 0.1 kg load, applied for 10 s. At least 10 random individual measurements were made. Finally, density measurement was conducted using the Archimedes method.
